# Lymph Node Ratio as a Prognostic Marker in Pancreatic Cancer Survival: A Systematic Review and Meta-Analysis

**DOI:** 10.7759/cureus.9597

**Published:** 2020-08-06

**Authors:** Uday Karjol, Ajay Chandranath, Pavan Jonnada, Sushma Cherukuru, Vinitha Annavarjula, Srinivas Ayyappa Morla

**Affiliations:** 1 Surgical Oncology, Kidwai Memorial Institute of Oncology, Bangalore, IND; 2 Pathology, AmPath Laboratories, Hyderabad, IND; 3 Oral and Maxillofacial Surgery, Surgical Oncology, Kidwai Memorial Institute of Oncology, Bangalore, IND; 4 Surgical Oncology, MNJ Institute of Oncology Regional Cancer Centre, Hyderabad, IND

**Keywords:** pancreatic cancer, lymph node ratio, positive lymph nodes, survival, metastatic lymph nodes

## Abstract

Introduction

The lymph node ratio (LNR) is defined as the ratio of number of positive lymph nodes to the total number of lymph nodes harvested during surgery. The objective of this article is to investigate the efficacy of LNR as a prognostic indicator of survival in pancreatic cancer patients who have undergone surgery by meta-analysis.

Methods

A systematic database search was performed in MEDLINE, Embase, and Google Scholar for relevant studies that reported LNR in pancreatic cancer. Two authors independently screened the relevant articles for selection and to extract data. All studies published in English up to April 2020 were obtained, and a total of 17,128 node-positive patients in 14 studies were included in this meta-analysis. RevMan software 5.3 (Cochrane Collaboration, the Nordic Cochrane Centre, Copenhagen, Denmark) was used for conducting all statistical analyses.

Results

This meta-analysis demonstrated that LNR > 0.2 significantly correlated with worse survival (hazard ratio [HR]: 1.84; 95% CI: 1.74-1.94; p ≤ 0.00001) in node-positive pancreatic cancer patients.

Conclusions

Our findings have demonstrated that a higher LNR is a predictor of poor survival and that LNR serves as an independent prognostic marker for assessing survival using a cut-off of 20%.

## Introduction

Pancreatic cancer with almost as many deaths as cases is the seventh leading cause of cancer death in both males and females [1]. It ranks as the second most common cause of death among all gastrointestinal malignancies [2]. Because of its advanced nature at the time of presentation, only a small proportion of patients are candidates for upfront resection. The actuarial five-year survival ranges only from about 15% to 25% even in patients who underwent resection [3,4].

Many histopathological factors were studied to correlate with prognostic outcomes in pancreatic adenocarcinoma. These factors include size of the tumor, number of lymph nodes (LNs), tumor grade, stage, and margin status of the resected specimen. LN metastasis is considered as an important factor for predicting overall survival and disease-free survival in non-metastatic pancreatic cancer patients who underwent surgery [5]. LN status acts as an important indicator of survival and recurrence in patients who have undergone resection for pancreatic cancer. The assessment of LN metastasis in pancreatic cancer is accomplished by the tumor node metastasis (TNM) staging system. The seventh edition of the American Joint Committee on Cancer (AJCC) classification system defined N1 as the presence of any number of positive regional LNs, whereas in the eighth edition of AJCC nodal classification was further categorized on the number of positive nodes [6].

Prognostic impact of lymph nodal metastasis can be assessed by using various methods such as the presence of positive LNs, total number of LNs, ratio of positive to total LNs, and also ratio of positive to negative LNs [7]. It should be noted that considering the number of positive LN involved to categorize nodal disease and to predict prognosis has been inaccurate. This may be due to the possibility of inadequate histopathological examination or incomplete lymphadenectomy, thereby missing on the actual positive LN count and thus resulting in stage migration [4]. Riediger et al. demonstrated that patients with one involved LN had same survival as patients with negative LN and that the prognosis of patients with one or no involved LN was significantly better than those with two or more positive LNs [3]. Moreover, it has also been demonstrated that there has been improvement in survival with increase in the number of negative LNs. All these results create uncertainty in prognostic risk stratification in node-positive pancreatic cancer patients who underwent surgical resection.

In recent times, LN ratio (LNR) is being evaluated as a prognostic indicator in several of the published studies apart from just using the number of positive LN [2-4,7-18]. The LNR is defined as the ratio of metastatic to the total number of harvested LNs, and it has emerged as an indicator of cancer-specific survival in recent years. The importance of LNR has been studied in various other solid malignancies and has been shown to be of significant prognostic value in rectal cancer survival in a recent meta-analysis [8].

In this study, using a meta-analysis, we have evaluated the prognostic influence of LNR on survival in pancreatic cancer patients who underwent surgery.

## Materials and methods

Search strategy

A systematic search was conducted on MEDLINE, Embase, and Google Scholar databases for all the articles published before April 2020. The search for articles was conducted by Medical Education Subject Headings (MeSH) keywords "LN" AND "ratio" AND "pancreatic cancer" OR "pancreatic carcinoma" AND "node positive" OR "metastatic LN". The search for articles and their reporting was conducted according to the Preferred Reporting Items for Systematic Reviews and Meta-Analyses (PRISMA) guidelines.

Study selection

By a comprehensive computer-based search, all the articles showing an association between LNR and survival of pancreatic cancer were identified. Two authors (P.J. and A.C.) assessed the titles and abstracts independently. Reference list of articles was scanned for similar additional articles. Assessment for eligibility was done and any discrepancy was resolved through discussion. Studies that met the following criteria were included: those published in English, studies on clinical trials, studies showing an association between survival and LNR and studies with quantitative outcome data after multivariate analysis (hazard ratio [HR] for survival). The exclusion criteria were failure to extract data from published articles, studies with republished data, publications in the form of meeting abstracts, comments, editorials, review articles, and those without reported outcomes.

Data extraction

Two authors (P.J. and A.C.) retrieved the necessary data from the screened full-text articles. From all the included studies, data retrieved included the following: basic study information including first author, publication year, study design, study setting, study duration, data sources, and multivariate adjustments; basic patient characteristics including age, gender, treatment, and survival periods; and comparative outcomes including HR for survival.

Quality assessment

Two authors (P.J. and A.C.) independently evaluated each of the studies using the Newcastle-Ottawa scale. The information of the studies included in shown in Table 1 [2-4,7,9-18]. The study was designated to be of poor quality if it did not meet more than one of the criteria in the selection domain, no score in the compatibility domain, and did not meet more than one of the criteria in the outcome domain. Any difference of opinion between the reviewers was resolved by consensus.

**Table 1:** Characteristics of the included studies SCT/RT, systemic chemotherapy/radiation therapy; NOS, Newcastle-Ottawa Scale; LNR, lymph node ratio; PCS, prospective cohort study; NA, not available; RCS, retrospective cohort study; ACT, adjuvant chemotherapy; ACRT, adjuvant chemoradiotherapy

Author name	Year	Study design	Sample size	SCT/RT	Average number of lymph nodes	Median follow-up/survival (months)	NOS	LNR stratification
Pawlik et al. [9]	2017	PCS	905	NA	17	24/17.4	7	0, 0-0.199, 0.2-0.399, >0.4
Riediger et al. [3]	2009	PCS	182	NA	16	NA/18	7	≤0.2, >0.2, ≤0.3, >0.3
Bhatti et al. [10]	2010	RCS	84	ACT	9	NA/22	6	0, 0-0.199, 0.2-0.299, >0.3
Murakami et al. [11]	2010	RCS	119	ACT	28		7	0, 0-0.99, 0.1-0.99, >0.2
Torre et al. [4]	2011	RCS	101	ACRT	19.8	NA/19	7	0, 0-0.199, 0.2-0.399, >0.4
Sanjay et al. [2]	2012	RCS	51	NA	20	NA/13	8	0, 0-0.199, >0.2
Torre et al. [12]	2014	RCS	192	ACRT	15	40/27	6	0, 0-0.199, 0.2-0.399, >0.4
Yamamoto et al. [13]	2014	RCS	56	ACT	27	NA/25	7	<0.2, ≥0.2
Liu et al. [14]	2014	RCS	167	NA	10	12/11.5	7	<0.4, >0.4
Zhan et al. [15]	2015	RCS	83	NA	8	26	7	≤0.2,>0.2
Mirkin et al. [16]	2017	RCS	14007	ACRT	15	NA	8	0, ≤0.2, 0.2-0.4, 0.4-0.8, >0.8
You et al. [7]	2019	PCS	351	ACT	18	NA/31	7	0, ≤0.2, 0.2-0.4, >0.4
Showalter et al. [17]	2011	PCS	445	ACT	9	NA/18	7	0, ≤1.5, 1.5-3.3
Robinson et al. [18]	2011	RCS	385	ACT	19	NA	7	0, ≤1.5, >1.5

Statistical analysis

The statistical analysis was performed using the RevMan software, version 5.3 (Cochrane Collaboration, the Nordic Cochrane Centre, Copenhagen, Denmark). Continuous variables were analyzed by the HR, and 95% CI was recorded. Heterogeneity was assessed using χ^2^ and I^2^ tests. I^2^ of 25%, 50%, and 75% represent low, moderate, and high heterogeneity, respectively. Studies with p < 0.1 and I^2^ > 25% indicated heterogeneity. The fixed-effects model was used with p > 0.10 and I^2^ < 25% for analysis. A random-effects model was used to estimate the pooled HR if significant heterogeneity existed in the fixed-effects model. The z-test was used to determine the pooled HR, and the significance was set to reject the null hypothesis at p < 0.05. Funnel plots were mapped out to investigate possible bias.

## Results

Studies included

**Figure 1:**
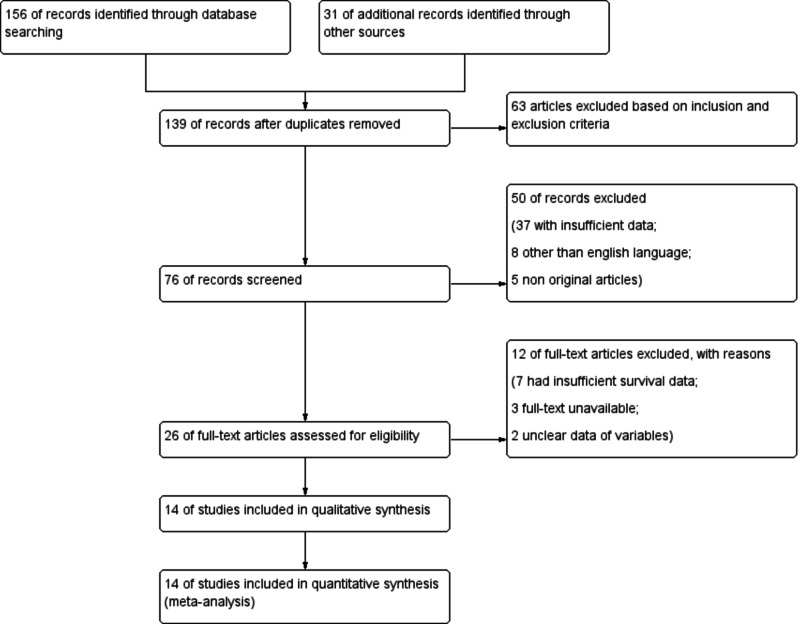
PRISMA flow chart showing study selection PRISMA, Preferred Reporting Items for Systematic Reviews and Meta-Analyses

A total of 14 potentially relevant articles were identified with our predefined search strategy. After excluding duplicates, 139 articles were identified. Based on inclusion and exclusion criteria and following the screening of titles and abstracts, 63 studies were excluded. The reviewers identified 26 studies for a full-text review. Of these, 14 studies were analyzed using meta-analysis after the exclusion of 12 studies (Figure 1). The quality of articles as assessed by the Newcastle-Ottawa score was by and large acceptable. The main characteristics of the included studies are provided in Table 1.

Results

In our meta-analysis study, we examined the effect of LNR on survival. Data were extracted from 14 studies, and the outcome regarding survival was assessed. The mean number LN retrieved in our study was 15.

**Figure 2:**
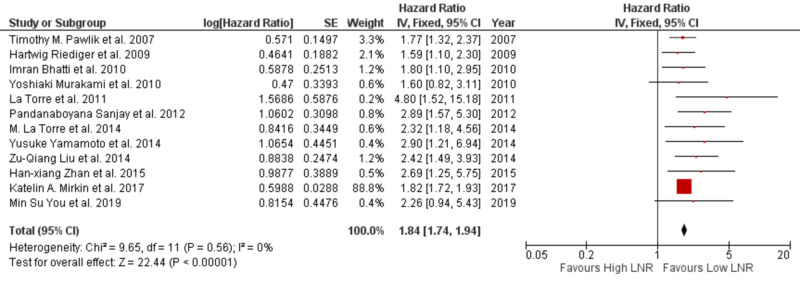
Forest plot showing LNR and survival SE, standard error; CI, confidence interval; LNR, lymph node ratio

Pooled HR and their 95% confidence interval (CI) were calculated for survival using the fixed-effects model. The results of our analysis revealed high LNR correlated with a poor survival. In I^2^ studies when a cut-off for LNR used was ≤0.2 and >0.2, the pooled HR was 1.84 (95% CI: 1.74-1.94) for survival, with a statistically significant p-value of <0.00001. The heterogeneity in our study was low (I^2^ = 0%; p = 0.56) on fixed-effects model, as shown in Figure 2.

**Figure 3:**
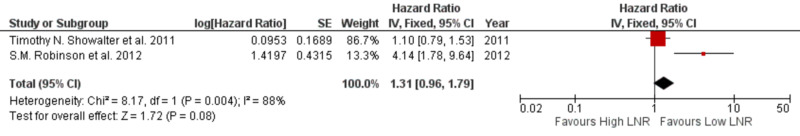
Forest plot showing LNR and survival SE, standard error; CI, confidence interval; LNR, lymph node ratio

In two studies, the cut-off for LNR used was ≤0.15 and >0.15. The pooled HR was 1.31 (95% CI: 0.96-1.79; p = 0.08) for survival. The heterogeneity in this group was high (I^2^ = 88%; p = 0.004) on fixed-effects model, as shown in Figure 3.

Publication bias

**Figure 4:**
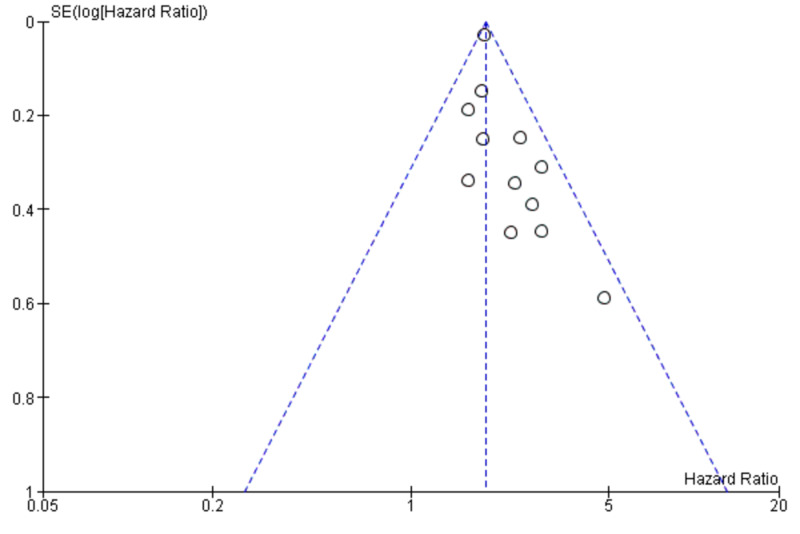
Funnel plot showing LNR (0.2) and survival SE, standard error; LNR, lymph node ratio

**Figure 5:**
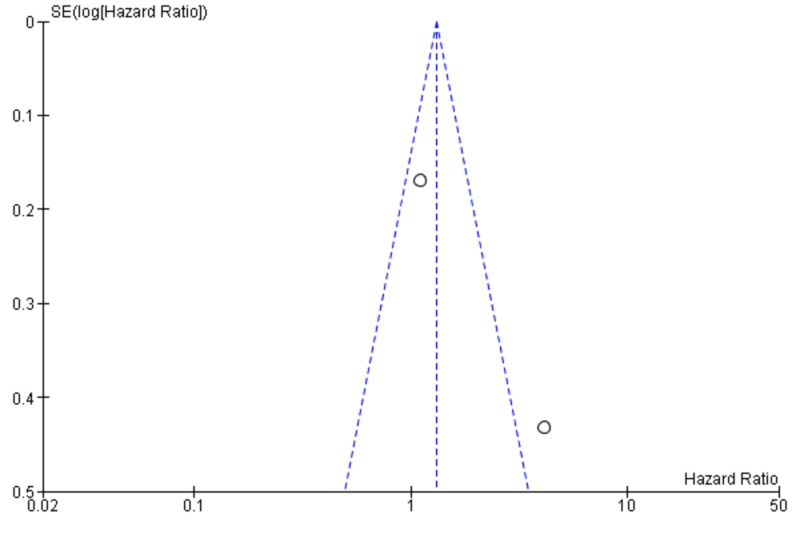
Funnel plot showing LNR (0.1) and survival SE, standard error; LNR, lymph node ratio

The publication bias of the included studies was evaluated by funnel plots. No visual publication bias was established, as shown in Figures 4, 5 [2-4,7,9-18]. This indicated that the publication bias was small in the current meta-analysis.

## Discussion

Long-term survival is achieved in exceptionally few patients of pancreatic cancer, and the only potential curative option in pancreatic adenocarcinoma is complete surgical resection. The presence of positive LN is deemed as a predictor of poor survival in several studies [3]. At the same time, it should be noted that nodal disease alone did not predict survival [10]. This may be due to inadequate histopathological examination or incomplete lymphadenectomy, or possibly due to stage migration [4]. These ambiguities in the prognostic impact of positive LN have led to the investigation of LNR in pancreatic cancer patients who underwent surgical resection. Our meta-analysis has proved that LNR is an independent prognostic factor of survival of pancreatic cancer patients.

The minimum number of LN to be harvested to evaluate the lymph nodal status of the patient who has undergone resection for pancreatic cancer as per the study conducted using the Surveillance, Epidemiology, and End Results (SEER) database is 12 nodes. However, the median number of nodes examined in their study is only 7 (range: 0-90). Slidell et al. showed that as the number of positive nodes increased, prognosis worsens. They also demonstrated that patients with one to three involved nodes had similar survival and, when compared to those patients with four to seven nodes, had better survival [19]. Liu et al. showed that patients with three or more involved LNs had worse survival in relation to those with less than three nodes [14]. This ambiguity has led investigators to search for an alternative that could effectively predict prognosis.

The recent literature has evidenced the beneficial role of LNR to reduce understaging and to explore prognostic significance in multiple gastrointestinal malignancies. LNR systematically evaluates and integrates both the number of positive nodes and the total number of LNs harvested [14]. LNR provides not only the number of involved nodes but also the adequacy of the LN yield. This could significantly influence the effect of LN status on survival of patients with resected cancer.

Many articles have used multiple cut-off of LNR in order to study their prognostic significance. There existed significant heterogeneity when studies using an LNR of 0.15 are included. Pooled analysis of our data demonstrated better survival in patients with low LNR as compared to a high value. We have mainly used an LNR of 0.2 as a cut-off, which has been validated in several studies [2-4,7,9-16]. The low heterogeneity in our studies further makes the result more noteworthy.

The merits of this meta-analysis are the precision of estimates that are based on a large dataset. This meta-analysis included 14 studies involving 17,128 resected pancreatic cancer patients. The statistical power of the study is satisfactory enough for our results. One more strength of this meta-analysis is the precision of LNR-specific estimates, confirming the stratifying cut-off of 20%. Finally, the other strength of our meta-analysis is the minimal heterogeneity between studies and their subgroups, which enhances the robustness of the results.

The findings of our study should be interpreted within the context of both the effectiveness and limitations of a study-level meta-analysis of heterogeneous studies. Therefore, a large cohort study or an individual patient data meta-analysis is required to affirm our results and establish the inconsiderable differences. There could be obligatory selection bias owing to the retrospective nature of the included studies. It should be that there are no randomized controlled trials on this topic in the literature that need to be mentioned.

## Conclusions

Our meta-analysis reviewed the current research addressing the prognostic role of LNR in assessing survival in pancreatic cancer patients who underwent surgical resection. Our findings have demonstrated that a higher LNR is a predictor of poor survival. Additionally, our study has demonstrated that LNR is an independent prognostic marker for assessing survival using cut-off of 20%. We conclude that the LNR could provide answers for the hiatus in the current nodal staging of the TNM staging system.
